# Similarly efficacious anti-malarial drugs SJ733 and pyronaridine differ in their ability to remove circulating parasites in mice

**DOI:** 10.1186/s12936-022-04075-z

**Published:** 2022-02-16

**Authors:** Arya SheelaNair, Aleksandra S. Romanczuk, Rosemary A. Aogo, Rohit Nemai Haldar, Lianne I. M. Lansink, Deborah Cromer, Yandira G. Salinas, R. Kiplin Guy, James S. McCarthy, Miles P. Davenport, Ashraful Haque, David S. Khoury

**Affiliations:** 1 QIMR Berghofer Medical Research Institute, 300 Herston Road, Herston, Brisbane, QLD 4006 Australia; 2 Infection Analytics Program, Kirby Institute for Infection and Immunity, UNSW Sydney, Sydney, 2052 Australia; 3 Department of Microbiology and Immunology, Peter Doherty Institute for Infection and Immunity, University of Melbourne, 792 Elizabeth Street, Parkville, VIC 3000 Australia; 4 Department of Infectious Diseases, Peter Doherty Institute for Infection and Immunity, University of Melbourne, 792 Elizabeth Street, Parkville, VIC 3000 Australia; 5 College of Pharmacy, University of Kentucky, Lexington, KY USA; 6 Victorian Infectious Diseases Service, Royal Melbourne Hospital, Parkville, VIC 3000 Australia

**Keywords:** SJ733, Pyronaridine, Artesunate, *Plasmodiumberghei*, Parasite clearance

## Abstract

**Background:**

Artemisinin-based combination therapy (ACT) has been a mainstay for malaria prevention and treatment. However, emergence of drug resistance has incentivised development of new drugs. Defining the kinetics with which circulating parasitized red blood cells (pRBC) are lost after drug treatment, referred to as the “parasite clearance curve”, has been critical for assessing drug efficacy; yet underlying mechanisms remain partly unresolved. The clearance curve may be shaped both by the rate at which drugs kill parasites, and the rate at which drug-affected parasites are removed from circulation.

**Methods:**

In this context, two anti-malarials, SJ733, and an ACT partner drug, pyronaridine were compared against sodium artesunate in mice infected with *Plasmodium berghei* (strain ANKA). To measure each compound’s capacity for pRBC removal in vivo, flow cytometric monitoring of a single cohort of fluorescently-labelled pRBC was employed, and combined with ex vivo parasite culture to assess parasite maturation and replication.

**Results:**

These three compounds were found to be similarly efficacious in controlling established infection by reducing overall parasitaemia. While sodium artesunate acted relatively consistently across the life-stages, single-dose SJ733 elicited a biphasic effect, triggering rapid, partly phagocyte-dependent removal of trophozoites and schizonts, followed by arrest of residual ring-stages. In contrast, pyronaridine abrogated maturation of younger parasites, with less pronounced effects on mature parasites, while modestly increasing pRBC removal.

**Conclusions:**

Anti-malarials SJ733 and pyronaridine, though similarly efficacious in reducing overall parasitaemia in mice, differed markedly in their capacity to arrest replication and remove pRBC from circulation. Thus, similar parasite clearance curves can result for anti-malarials with distinct capacities to inhibit, kill and clear parasites.

## Background

Malaria is caused by protozoan parasites from the genus *Plasmodium,* which cyclically invade, replicate within and rupture out of red blood cells (RBC) in the host. There were an estimated > 200 million cases of malaria and > 400,000 deaths annually between 2015 and 2020 [1]. Artemisinin-based combination therapy (ACT) has been the recommended first-line treatment for malaria for several years [2]. However, emergence of parasite resistance to artemisinin-based combinations is threatening progress towards malaria elimination [3]. This has prompted development of both new artemisinin-based combinations and new candidate anti-malarial drugs.

The kinetics of circulating parasitized red blood cells (pRBC) after anti-malarial drug treatment is commonly referred to as the “parasite clearance curve” (PCC). It has been a useful proxy for determining parasiticidal and clinical efficacy of drug candidates [4]. However, ex vivo culture of pRBC from *Plasmodium falciparum*-infected, artesunate-treated human volunteers has recently been used to demonstrate that parasites are rendered non-viable well before any detectable change in the PCC [5]. This revealed a disconnect, and possibly an under-estimation, of anti-malarial drug efficacy when the principal metric is the PCC. It has recently been proposed that the PCC is a composite of parasiticidal/parasitostatic drug effects and removal of pRBC [4]. Hence, the hypothesis here is that two highly effective anti-malarials can reduce total parasite numbers by the same magnitude, yet differentially remove and/or arrest the development of circulating parasites.

Previously, experimental and mathematical modelling approaches have been developed for quantifying parasite replication and pRBC removal in mice [6–9]. This involved flow cytometric tracking over time of a cohort of fluorescently-labelled pRBC [10], thereby permitting observation of maturation, schizogony, and loss of the first generation of parasites, and emergence of subsequent generations. Using this approach revealed instances where immune responses controlled parasite numbers without removing pRBC [6, 9], and where two licensed anti-malarial drugs, sodium artesunate and mefloquine, permitted drug-affected parasites to persist in circulation [7, 8]. Moreover, mefloquine-affected pRBC were removed more slowly than artesunate-affected pRBC, suggesting not all drugs are equivalent at inducing removal of drug affected pRBCs.

Here, two important anti-malarial compounds, SJ733 and pyronaridine were examined [11, 12]. SJ733 is an inhibitor of *P. falciparum* ATP4, a sodium-proton antiporter. Recent first-in-human trials revealed rapid, though un-sustained, disappearance of parasites from circulation with a clearance half-life of  ~ 4–7 h [13], supporting SJ733 as a possible partner drug for longer-acting anti-malarial drugs. SJ733 was also reported to be amongst a small number of anti-malarial drugs known for their ability to increase the rigidity of pRBC, potentially increasing susceptibility to splenic removal [14, 15]. Pyronaridine is a potent anti-malarial with a long history of clinical use, particularly in China. It has a long half-life [12], has been reported to cause fast reduction in pRBC numbers in mice [16], and has faster in vitro activity compared with many other long acting anti-malarial drugs [17]. Pyronaridine was recently developed as a long-lasting partner drug in ACT [1, 18, 19]. Here, *Plasmodium berghei* (strain ANKA) infection of mice was used to examine the in vivo dynamics of pRBC killing and removal after drug treatment. While SJ733 and pyronaridine similarly reduced total parasite numbers by 24 h post-treatment, pRBC removal rates and killing kinetics were distinct. SJ733 rapidly removed mature stages, leaving younger ring-stages to circulate, while pyronaridine rapidly inhibited ring-stages, allowing a portion of mature parasites to complete one transition to the next generation of RBC, while only modestly increasing RBC removal.

## Methods

### Experimental mice and blood-stage *Plasmodium* infection

C57BL/6 J mice were purchased from the Animal Resource Centre (Perth, Australia). All mice were female between 6 and 12 weeks of age and were maintained under conventional conditions. This study was carried out in strict accordance with guidelines from The National Health and Medical Research Council (NHMRC) of Australia. All animal procedures and protocols were approved (A02-633 M and A1503-601 M) and monitored by the QIMR Berghofer Medical Research Institute Animal Ethics Committee. *Plasmodium berghei* (strain ANKA) parasites, constitutively expressing high-levels of enhanced Green Fluorescence Protein (GFP), were sourced and used as previously reported [9, 20, 21]. *Plasmodium berghei* parasites were used after defrosting cryopreserved infected blood and a single in vivo passage in C57BL/6 J mice. RBCs were collected from passage mice by cardiac puncture and used to infect via lateral tail vein injection.

### Drug preparation and administration

Saline was used a negative control for all experiments involving drug treatment. Sodium artesunate (Guilin Pharmaceutical or sourced from J. Mohrle) was used as a positive control and was prepared according to the manufacturer’s protocol by dissolving in 5% sodium bicarbonate solution, diluting in 0.9% saline (Baxter) to a final concentration of 5 mg/ml. Mice were treated via intraperitoneal injection (1 mg per 200 μl dose). SJ733 and pyronaridine (AssayMatrix) were dissolved in a drug formulation consisting of ethyl alcohol, propylene glycol, Carbowax^®^ PEG 400, phosphate buffered saline (PBS) (pH 7.4) and 2-hydroxypropyl-β-cyclodextrin (HβCD). SJ733 solution was sonicated for 15 min and pyronaridine solution was placed on a roller for 15 min and then sonicated for 5 min in order to yield clear solutions. Both drugs were administered to mice by oral gavage. Clodronate liposomes (Liposoma; www.clodronateliposomes.com) were used for depleting macrophages, as previously described [6–8], and were injected three days before infections with a single i.v. injection (200 μl, lateral tail vein).

### RBC adoptive transfer

Adoptive transfer of fluorescently-labelled RBC was performed as previously described [6, 9]. RBCs were collected from infected mice by cardiac puncture, washed twice in Ca^2+^- and Mg^2+^-free PBS, and stained with viable cell dye CellTrace™ Far Red (CTFR; Thermo Fisher). Briefly, 50 μg CTFR was dissolved for ten minutes in 25 μl tissue culture grade dimethyl sulphoxide. This was added to 5 ml of resuspended RBC in Ca^2+^- and Mg^2+^-free PBS. RBC were stained in the dark, at room temperature with constant rolling for 15 min, and washed twice in 10 × volumes of Ca^2+^- and Mg^2+^-free PBS. CTFR-labelled RBCs were resuspended in 2 ml Roswell Park Memorial Institute 1640 (RPMI) medium per donor mouse and injected in 200 μl volumes to recipient mice via lateral tail vein.

### Flow cytometry of RBC

Established flow cytometric methods [6–9] were employed to track the first generation of pRBC, termed Gen_0_ pRBC, and to distinguish these from subsequent generations of pRBC (termed Gen_1+_), which were identified as un-labelled, endogenous RBC infected with the merozoite progeny of Gen_0_ pRBC. This staining approach also permitted assessment of parasite life-stages (ring, trophozoite and schizont), as previously reported [6–9]. Briefly, a single drop of blood from a tail bleed was diluted and mixed in 200 μl of RPMI medium containing 5 U/ml heparin sulphate. Diluted blood was simultaneously stained for 30 min in the dark at room temperature with cell-permeant RNA/DNA stain, Syto84 (5 μM; Life Technologies) and with DNA stain, Hoechst 33342 (10 μg/ml; Sigma). Staining was quenched with 10 volumes of ice-cold RPMI medium, and samples immediately analysed by flow cytometry using an LSRII Fortessa analyser (BD Biosciences) and FlowJo software (Treestar, CA, USA). FSC-Area (FSC-A) and FSC-Height (FSC-H) were used to exclude doublets (the result of two cells being detected simultaneously by the flow-cytometer). FSC-A and side scatter (SSC-A) were used to distinguish RBCs from debris and white blood cells on the basis of size and granularity. Infected RBCs were detected as co-expressing Hoechst 33342 and Syto84, with expression of either one alone being insufficient to identify the presence of parasites. Adoptively-transferred Gen_0_ pRBCs were readily distinguished from endogenous RBC, and importantly from Gen_1+_ pRBC, by CTFR-labelling.

### Ex vivo culture

Tail blood was collected from mice into 1.5 ml Eppendorf tubes with culture medium consisting of 80% RPMI, 20% fetal bovine serum (FBS) and 1/1000 heparin and 200 μl was plated per well in 96 well plates. Plates were covered then flushed for 30 s with 5% CO_2_, 5% O_2_, 90% N_2_ in a closed secondary container which was then incubated at 37 °C.

### Statistical analysis

All comparisons of groups were performed using a t test. A one-way analysis of variance (ANOVA) was used to compare means from more than 2 groups. Multi-way ANOVA was used to compare groups when there were multiple variables, in particular to assess whether there was an interaction between clodronate treatment and drug treatment. All data analysis was performed in MATLAB version R2020a. All summary statistics in the text are provided as arithmetic mean and standard deviations. A logistic growth model was used to estimate the viable parasites 24 h after treatment, by fitting growth of parasitaemia. The model has the form,

$$P\left( t \right) = \frac{{C_{0} P_{0} e^{{g\left( {t - t_{0} } \right)}} }}{{C_{0} + P_{0} \left( {e^{{g\left( {t - t_{0} } \right)}} - 1} \right)}}$$

where $P$ is the parasitaemia at time, $t$, ${C}_{0}$ is the saturating level of parasitaemia (as time approaches infinity), ${P}_{0}$ is the parasitaemia at a specified initial time ${t}_{0}$ and $g$ is the initial growth rate (i.e. at time ${t}_{0}$) of parasites. The constant $e$ is the mathematical constant. Logistic growth, rather than simple exponential growth, was used to model this data because growth of parasitaemia is saturating, likely due to target cell limitation [22]. The built in function *lsqnonlin* was used to fit this model to the growth of parasitaemia after treatment in MATLAB. In addition, the parasite clearance half-lives were estimated by fitting each individual mouse decline curve on a log-scale with a linear regression model, using the function *fitlm* in MATLAB. The half-life was calculated as log(2) divided by the slope of the log-linear decline.

## Results

### SJ733 and pyronaridine are similarly efficacious at reducing parasitaemia in *P. berghei*-infected mice

**Fig. 1:**
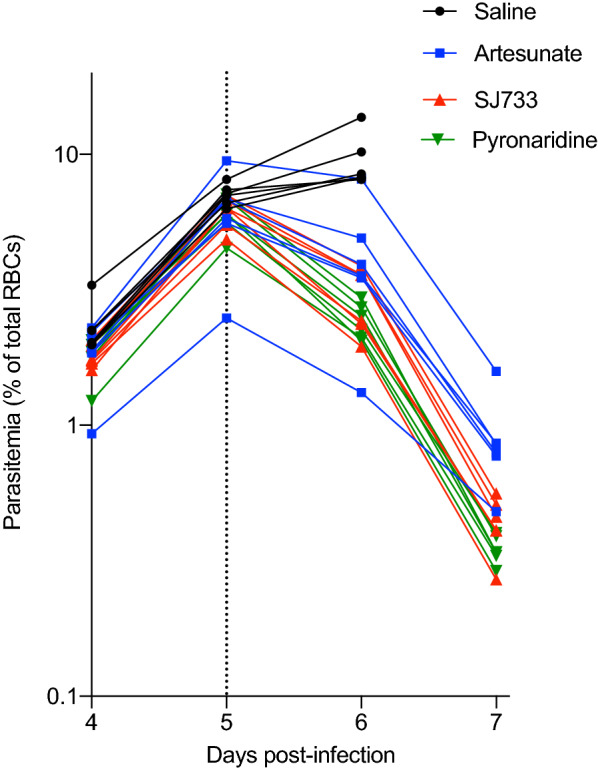
SJ733 and pyronaridine are similarly efficacious at reducing parasitaemia in 5-day *P. berghei*-infected mice. C57BL/6 J mice (n = 6/group) were infected with blood-stage *P. berghei* (strain ANKA) parasites, individually monitored for blood parasitaemia daily from day 4 post-infection (p.i.), and treated with single dose anti-malarials SJ733, pyronaridine or sodium artesunate on day 5 p.i. (indicated with vertical dotted line). Control saline treated mice were ethically euthanised due to severe symptoms on day 6 p.i

Firstly, it was confirmed that SJ733 and pyronaridine rapidly reduce circulating parasitaemia in mice. C57BL/6 J mice, 5 days after infection with *P. berghei*, generally exhibited a parasitaemia exceeding ~ 2% (Fig. 1). Liver damage is apparent by this stage of infection, although severe neurological and pulmonary complications are yet to develop [23]. Mice were treated with a single dose of SJ733 (200 mg/kg), pyronaridine (10 mg/kg, doses established from in vivo titration studies), or the rapidly acting drug, sodium artesunate (50 mg/kg) as a positive control, and fold-reductions in parasitaemia determined 24 h after treatment relative to saline controls. All control mice (i.e., saline treated mice) exhibited clinical scores requiring ethical euthanasia at day 6 (as expected for this severe and lethal model [24]), whereas none of the artesunate, SJ733 or pyronaridine-treated mice showed neurological complications over this time period. Parasitaemia following treatment with sodium artesunate declined 1.6 ± 0.25 fold over the first 24 h (Fig. 1). The fold-decline for SJ733 and pyronaridine was 2.1 ± 0.38 and 2.5 ± 0.26, respectively. This confirmed anti-malarial drug efficacy for SJ733 and pyronaridine in this experimental malaria model. Further, these results demonstrate that at the doses used, these two drugs exhibited greater reductions in pRBC over the first 24 h than sodium artesunate (P = 0.0079 and P = 0.0003), despite the very high dose of sodium artesunate [8].

### SJ733 substantially increases pRBC removal

**Fig. 2:**
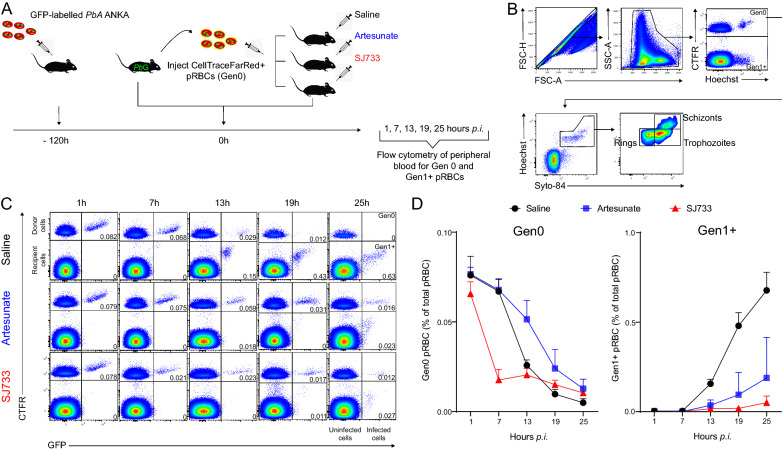
SJ733 rapidly removes blood-stage parasites from circulation. **A** Schematic showing C57BL/6 J mice (n = 6/group) administered CTFR^+^ RBC harbouring *P. berghei*-GFP (termed Gen_0_), treated immediately with control saline, sodium artesunate or SJ733, and peripheral blood monitored individually at times indicated by flow cytometry to monitor Gen_0_ pRBC and emergence of Gen_1+_ pRBC. **B** Flow cytometric gating strategy for identifying Gen_0_ and Gen_1+_ pRBC and assessing parasite life-stages. **C** Representative FACS plots for total RBC over time, showing the dynamics of Gen_0_ and Gen_1+_ pRBC. **D** Summary of Gen_0_ and Gen_1+_ pRBC dynamics expressed as a percentage of total RBC. Error bars represent the standard the standard error of the mean

Since the decline in parasitaemia is a composite measure of pRBC removal and parasite replication, the established RBC adoptive transfer approach [6–9] was used to directly measure whether SJ733 had altered the rate of pRBC removal from circulation. Mice were infused with fluorescently-labelled RBC containing *P. berghei*-GFP parasites, and were immediately treated with SJ733 (200 mg/kg), sodium artesunate (50 mg/kg) or control saline (Fig. 2A). Flow cytometric assessment of peripheral blood was then conducted to monitor the fate of fluorescent pRBC. Parasites observed in their original, fluorescent RBC were considered “Generation 0” (Gen_0_). Parasites observed in non-fluorescent RBC, judged to have invaded new RBC, were termed “Generation 1 + ” (Gen_1+_) (Fig. 2A, B). In the absence of anti-malarial drugs, the expected gradual loss of Gen_0_ pRBC, due to maturation, rupture and phagocytic removal and concomitant emergence of Gen_1_ pRBC was observed. In contrast to previous studies [6–9], Gen_0_ pRBC were lost from circulation at a substantially higher rate after SJ733 treatment compared to saline controls, specifically over the first seven hours post-treatment, termed Phase I (Fig. 2C, D). By 7 h post-treatment,  ~ three-fold fewer Gen_0_ pRBC (32.2% $\pm$ 9.7) remained in circulation in SJ733-treated mice compared to either saline control mice (91.0% $\pm$ 4.6, P = 0.0002) or sodium artesunate-treated mice (94.2% $\pm$ 6.2, P = 0.0009) (Fig. 2C, D). Thus SJ733, in contrast to artesunate, substantially increased the rate of pRBC removal within the first 7 h after treatment.

After the initial 7 h period, Gen_0_ pRBC appeared to persist and plateau in SJ733-treated mice, which contrasted with the continued, gradual loss observed in control saline-treated mice, and with the previously reported persistence of drug-affected parasites in sodium artesunate-treated mice (Fig. 2C, D). Nevertheless, by 25 h Gen_0_ pRBC concentrations were similar between drug-treated groups. This indicated that although SJ733 induced a rapid Phase I of pRBC removal, a Phase II also occurred (7–25 h), in which Gen_0_ pRBC persisted longer than in control-treated mice (Fig. 2C, D). The biphasic dynamics for Gen_0_ pRBC after SJ733 treatment was distinct from that of sodium artesunate, illustrating differing drug effects on pRBC in vivo. Interestingly, the pRBC removal rate in the first 7 h was higher with SJ733 than other previously reported treatments or immunological interventions [6–9].

### SJ733 targets late-stage pRBC for rapid removal

**Fig. 3:**
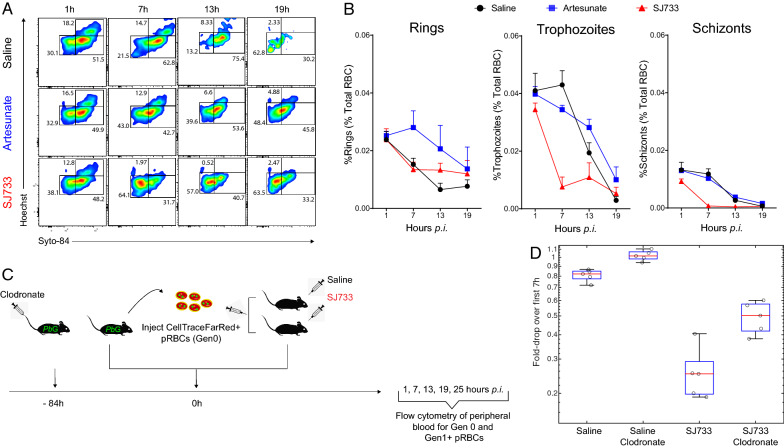
SJ733 targets mature life-stages for early removal, with a partial requirement for phagocytes. **A** Representative FACS plots showing life-stage gating for rings, trophozoites and schizonts in Gen_0_ pRBC over time. Mice (n = 6/group) were administered CTFR^+^ RBC harbouring *P. berghei*-GFP (termed Gen_0_), treated immediately with control saline, sodium artesunate or SJ733, and peripheral blood monitored individually at times indicated by flow cytometry. **B** Summary dynamics for Gen_0_ rings, trophozoites and schizonts expressed as a percentage of all RBC. **C** Schematic the experimental approach. Mice (n = 5/group) were phagocyte-depleted treated with clodronate liposomes 3 days prior to the transfer of CTFR^+^ RBC harbouring *P. berghei*-GFP. Mice were then immediately treated with SJ733 or saline control, and flow cytometric assessment of Gen_0_ and Gen_1+_ pRBC was performed at the times indicated. **D** Summary of drop in Gen_0_ pRBC levels over the first 7 h. Error bars represent the standard the standard error of the mean

The biphasic kinetics with which Gen_0_ pRBC were lost from circulation after SJ733 treatment suggested possible heterogeneous effects on parasites. Flow cytometric assessment of DNA and RNA content within pRBC permitted categorization into three stages of maturation [10]. At the time of transfer into mice, Gen_0_ pRBC exhibited a spectrum of maturation states (Fig. 3A). SJ733 treatment induced a pronounced loss of trophozoites and schizonts during Phase I (Fig. 3A, B), which contrasted with increases in these life-stages in control mice (P < 0.0001) (Fig. 3B). A two-point clearance half-life revealed that late-stage parasites were lost with a net half-life of 3.0 $\pm$ 1.0 h during this window. These data indicate that the rapid removal of parasites induced by SJ733 treatment in Phase I is primarily the result of rapid removal of mature trophozoite and schizont forms.

The original murine study of SJ733 efficacy used mice that were splenectomized or phagocyte depleted via clodronate liposomes (in the humanized mouse model—NOD-scid IL2Rg^null^ mice infected with *P. falciparum*) and found that the rapid effect of SJ733 within the first 12 h after treatment was not strongly dependent upon the presence of a spleen or splenic phagocytes [11]. This was further explored here by determining the effect of clodronate liposomes on SJ733-mediated removal of Gen_0_ pRBC within 7 h after treatment. Firstly, consistent with previous reports [7, 8], after clodronate treatment alone Gen_0_ pRBC remained in circulation for longer (Fig. 3C, D), indicating the efficacy of the phagocyte depletion regimen used here. Next, it was found that although SJ733 caused a rapid loss of Gen_0_ pRBC in clodronate-treated mice, the magnitude of this effect was reduced (Fig. 3D, fold-drop in Gen_0_ over 7 h exhibited significant interaction between SJ733 treatment and clodronate in two-way ANOVA, P = 0.0042). Given that clodronate treatment removes splenic macrophages effectively [7, 8], the data reported here support only a partial role for these cells in rapid removal of SJ733-affected Gen_0_ pRBC from circulation.

### Parasiticidal efficacies of SJ733 and sodium artesunate are similar despite differences in pRBC removal

**Fig. 4:**
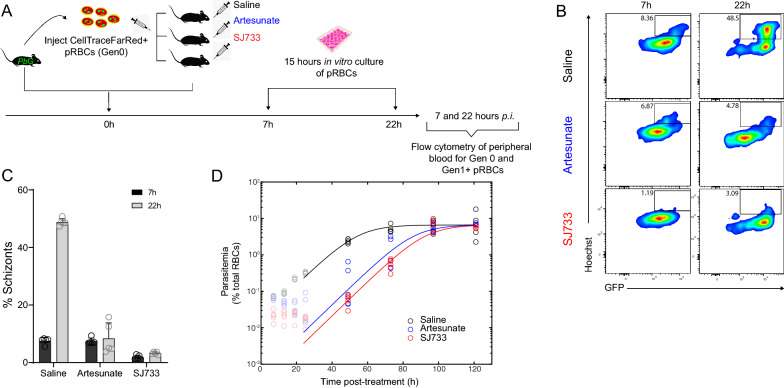
SJ733 arrests maturation and replication in ring-stage parasites. **A** Schematic showing that 7 h after Gen_0_ pRBC adoptive transfer into mice (n = 5/group) and SJ733, artesunate or control saline treatment, peripheral blood was cultured ex vivo to determine the capacity of remaining drug-exposed Gen_0_ pRBC to mature and undergo schizogony. **B** Representative FACS plots depicting schizogony with Hoechst33342 staining in Gen_0_ pRBC over 15 h of culture, starting at 7 h post-in vivo drug exposure. **C** Summary graph for individual mice, showing percentage of Gen_0_ pRBC that were schizonts before and after ex vivo culture. **D** A logistic growth model fit to saline-treated controls (black), 50 mg/kg, artesunate (blue), and 200 mg/kg, SJ733 (red)-treated infections. The model was used to estimate the number of viable parasites remaining 24 h after treatment

During Phase II after SJ733 treatment (Fig. 2D), more Gen_0_ pRBC remained compared to controls by 25 h (P = 0.0001, 17.4 ± 2.0% versus 6.9 ± 2.8% of the initial proportions at 1 h), suggesting SJ733 had impaired or inhibited Gen_0_ pRBC maturation (Fig. 3B). To determine maturation potential of Gen_0_ pRBC surviving Phase I, RBC were harvested at 7 h after SJ733 treatment and cultured ex vivo (Fig. 4A). This revealed the majority of residual parasites did not mature into schizonts over 15 h of ex vivo culture (5.4 ± 0.8% of SJ733-exposed parasites reached schizont stage, compared with 54.0 ± 0.7% for saline controls, P < 0.0001) (Fig. 4B, C), but were capable of partial maturation as evidenced by an increase in life-stage dependent GFP expression (Fig. 4B).

In vivo parasite regrowth was observed in SJ733-treated mice, similar to that seen after sodium artesunate treatment, which indicated that a proportion of parasites had progressed to schizogony after single-dose treatment with either drug (Fig. 4D). Modelling in vivo regrowth of parasitaemia suggested that SJ733 had reduced viable parasitaemia by 98.4% (95% CI 96.0, 99.3) of that observed in control mice 24 h after treatment. Similarly, a single dose of sodium artesunate reduced viable parasitaemia by 96.7% (95% CI 92.0, 98.7) of that in control mice. Therefore taken together, this data reveal that although SJ733 triggered a unique, rapid removal of late-stage pRBC, by slowing maturation and impairing schizogony, the overall effect on viable parasite numbers 24 h after treatment was similar for SJ733 and artesunate, with both treatments causing persistence of pRBC in Gen_0_.

### Pyronaridine abrogates maturation, increases removal of drug-affected ring-stages, and cures established *P. berghei* infection

**Fig. 5:**
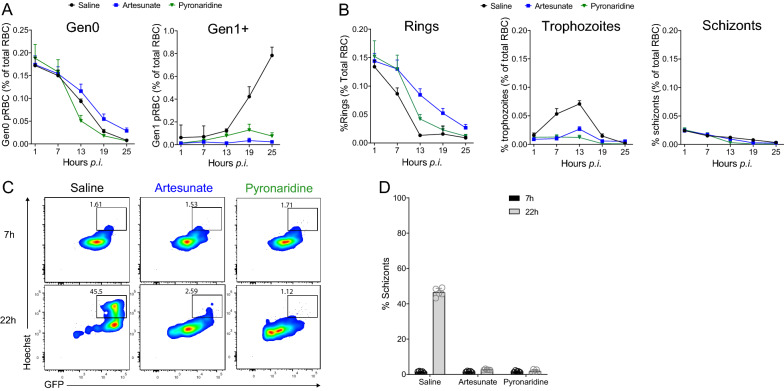
Pyronaridine arrests maturation in ring-stages and modestly increases pRBC removal. **A** Summary of Gen_0_ and Gen_1+_ pRBC dynamics expressed as a percentage of total RBC after single dose treatment with pyronaridine, artesunate or control saline (n = 5 mice/group). **B** Summary dynamics for Gen_0_ rings, trophozoites and schizonts expressed as a percentage of all RBC. **C** Representative FACS plots depicting schizogony with Hoechst staining, and GFP expression in Gen_0_ pRBC over 15 h of culture, starting at 7 h post-in vivo drug exposure. **D** Summary graph for individual mice, showing percentage of Gen_0_ pRBC that were schizonts before and after ex vivo culture. Error bars represent the standard the standard error of the mean

Pyronaridine showed a similar decline in parasitaemia over the first 24–48 h period compared to SJ733 (Fig. 1). Hence the effects of this drug on pRBC clearance and parasite killing was also examined. Following pyronaridine treatment, a modest increase in the loss of Gen_0_ pRBC compared to controls was apparent by 12 h post-infection (Fig. 5A). Despite the loss of Gen_0_ pRBCs at a similar or faster rate compared to untreated controls, ring-stage parasites persisted in the first 7 h after treatment compared with controls (Fig. 5B), suggesting that ring-stage parasites had been rapidly inhibited by pyronaridine. To test this, Gen_0_ pRBCs collected 7 h after in vivo treatment with pyronaridine were cultured in vitro*,* and, similar to artesunate, it was observed that maturation had been abrogated, with parasites in fact losing life-stage dependent GFP expression (54.6 ± 1.0% drop in the mean florescence intensity of GFP (GFP-MFI), P < 0.0001) (Fig. 5C, D). In contrast, parasites from control mice showed a 7.3 ± 0.35 fold increase in GFP-MFI over 15 h of in vitro culture. Since ring-stage development had been inhibited by 7 h in vivo treatment the authors inferred that the loss of pRBC in vivo at later time points was due to removal of ring-stage pRBC and estimated the half-life of removal of rings after pyronaridine treatment to be 4.0 ± 0.21 h (Fig. 5B). In contrast to rapid ring-stage inhibition, it was evident that a portion of trophozoites and schizonts were not inhibited from rupturing and producing Gen_1_ parasites up until 19 h post-treatment. Thus, while the majority of parasites remaining at 24 h after artesunate or SJ733 treatment were Gen_0_ pRBC, in mice treated with pyronaridine these were mostly Gen_1_ pRBC. This suggested pyronaridine exhibited rapid action on ring-stage parasites in vivo, but was less effective at rapidly blocking schizogony and subsequent invasion compared with artesunate and SJ733. Finally, no recrudescence of infection after single dose pyronaridine was observed. Thus, unlike other drugs tested, including sodium artesunate, mefloquine or SJ733, a single dose of pyronaridine cured a high inoculum *P. berghei* infection. Taken together, these results indicate that the longer-acting pyronaridine rapidly inhibits ring-stage maturation, accelerates their removal, and ultimately elicited single-dose cure in this lethal murine model.

## Discussion

In this study, a blood-stage *Plasmodium* infection model in mice was used to provide mechanistic detail on how the anti-malarial drugs SJ733 and pyronaridine induce rapid loss of pRBC in vivo. Having confirmed that these two drugs were no less effective than artesunate at reducing parasitaemia in this murine model, two parameters were examined in this study: firstly, the rate at which pRBC were removed from circulation after drug treatment, and secondly, parasite replicative fitness via the capacity to mature within RBC and to give rise to the next generation of pRBC. These two parameters cannot be readily inferred from longitudinal parasitaemia measurements alone.

Recent first-in-human trials indicated that SJ733 was well-tolerated, had a good safety profile, and caused a rapid, though unsustained, reduction in parasite numbers after a single-oral dose [13]. The in vivo mechanism of action inferred from the initial murine study, suggested that SJ733 affects all life-stages, and that rapid loss of pRBC occurs without a requirement for the spleen or phagocytes [11]. The data in the present study provides further detail, by revealing that SJ733 elicits its effect in two phases. During Phase I (0–7 h post-treatment), SJ733 caused rapid removal of late-stage pRBC. In contrast to the limited increase in removal of pRBCs observed previously with other compounds [7, 8], the present data has confirmed that pRBC removal can be substantially enhanced in vivo by the action of some compounds. During Phase II, after 7 h exposure to SJ733, residual ring-stage parasites were partially impaired from maturing into trophozoites and schizonts. In addition, while clodronate liposome-treatment did not entirely abrogate removal of late-stage pRBC, the efficacy of this process was reduced significantly. Clodronate has been shown to remove splenic macrophages more so than Kupffer cells in the liver [7, 8], suggesting that SJ733 may partially rely on host splenic macrophages to remove late-stage pRBC. This evidence for a partial role of phagocytosis must be interpreted with caution as it is difficult to discern from the lower overall clearance observed in clodronate treated mice. Together, the data presented here is consistent with SJ733 acting on all life-stages in vivo, but emphasises that the removal of drug-affected parasites is dramatically different for early and late-stages.

Interestingly, clearance of parasites after artesunate and SJ733 was rapid, the estimated drop in viable parasites (based on regrowth of parasitaemia) was greater than the drop in total circulating parasitaemia (Fig. 4D). This is consistent with observations that parasites are killed by artesunate more quickly than they are removed by the host [5].

A limitation of the infection model employed in this study (*P. berghei* strain ANKA in C57BL/6 J mice), compared with a humanized mouse model of infection is that it was not possible to study a human parasite species, and drug inhibitory effect may vary between parasite species. However, given the aim of this study is to understand parasite clearance after drug treatment, it was reasoned that the use of mice with intact circulatory, immune and mononuclear phagocytic systems should be prioritized, which precluded the use of a humanized mouse model. Interestingly, the observation here of rapid splenic removal of drug-affected late-stage parasites is not a phenomenon that could be observed in human *P. falciparum* infection because late-stage parasites are sequestered in the microvasculature in human infection and so such rapid removal is not expected to occur. Thus, in humans the decline in parasitaemia would be expected to be governed predominately by ongoing development of some ring-stage parasites into trophozoites, which sequester and/or are removed by the spleen.

Pyronaridine induced a faster loss of pRBCs from circulation than artesunate and a rapid inhibition of parasite development, but failed to abrogate the appearance of the next generation of parasites. This combination of rapid clearance yet imperfect inhibition of schizogony and invasion is an interesting property of the drug. Similar to SJ733, pyronaridine induced a rapid loss of late-stage parasites; given next generation (Gen_1+_) parasites were observed after treatment, this may have occurred due to both rupture of schizonts as well as clearance of parasites. Pyronaridine also rapidly inhibited ring-stage parasites, and increased their clearance. This was surprising given that the reported mechanisms of action of pyronaridine are believed to be on trophozoite stages and formation of haemozoin [25]. In vitro studies suggested that haematin and pyronaridine interactions accelerate red cell lysis [25], and the authors speculate that the rapid loss of ring and late-stage parasites in this murine system may be partially due to enhanced lysis of RBCs. Pyronaridine is unique in its ability to cure established (i.e. day 5) *P. berghei* infection in mice. This is consistent with findings of numerous other murine studies (reviewed in [12]), where high cure rates were reported in mice treated with pyronaridine, even when compared with other long-acting compounds, an effect that is likely attributed to a combination of the long half-life of the drug and its low IC_50_ compared with other long-acting compounds.

The experimental models used in this study were designed to test basic questions of how pRBC numbers could be rapidly lowered during malaria. In this study, three anti-malarials producing similar declines in total parasitaemia after 24 h are demonstrated to achieve this by distinctly different underlying kinetics of growth inhibition and pRBC clearance. In the animal model with intact circulatory, immune and mononuclear phagocytic systems examined here, it was demonstrated that pRBC removal rates can be increased in vivo, as evidenced by SJ733, which accelerates late-stage pRBC removal, and pyronaridine which accelerates early-stage pRBC removal. These two drugs were therefore unlike sodium artesunate, which permits drug-affected parasites to remain in the circulation for longer, and did not substantially increase the rate of removal by host phagocytes [7, 8].

## Conclusions

In summary, in the murine infection model reported here, accelerated pRBC removal, either by lysis or splenic filtration, and abrogation of maturation and prolonged action in vivo, are major determinants of the clearance of parasitaemia after treatment, and that these kinetics vary considerably amongst compounds with different modes of action.

## Data Availability

All data will be made available upon reasonable request.

## References

[1] WHO Global Malaria Programme (2019). World Malaria Report 2019.

[2] Davis TM, Karunajeewa HA, Ilett KF (2005). Artemisinin-based combination therapies for uncomplicated malaria. Med J Aust.

[3] Menard D, Fidock DA (2019). Accelerated evolution and spread of multidrug-resistant *Plasmodium falciparum* takes down the latest first-line antimalarial drug in southeast Asia. Lancet Infect Dis.

[4] Khoury DS, Zaloumis SG, Grigg MJ, Haque A, Davenport MP (2020). Interdisciplinary Approaches to Malaria C. Malaria parasite clearance: what are we really measuring?. Trends Parasitol.

[5] Rebelo M, Pawliw R, Gower J, Webb L, Mitchell H, Pava Z (2021). Parasite viability as a superior measure of antimalarial drug activity in humans. J Infect Dis.

[6] Akter J, Khoury DS, Aogo R, Lansink LIM, SheelaNair A, Thomas BS (2019). *Plasmodium*-specific antibodies block in vivo parasite growth without clearing infected red blood cells. PLoS Pathog.

[7] Aogo RA, Khoury DS, Cromer D, Elliott T, Akter J, Fogg LG (2018). Quantification of host-mediated parasite clearance during blood-stage *Plasmodium* infection and anti-malarial drug treatment in mice. Int J Parasitol.

[8] Khoury DS, Cromer D, Elliott T, Soon MSF, Thomas BS, James KR (2017). Characterising the effect of antimalarial drugs on the maturation and clearance of murine blood-stage *Plasmodium* parasites in vivo. Int J Parasitol.

[9] Khoury DS, Cromer D, Akter J, Sebina I, Elliott T, Thomas BS (2017). Host-mediated impairment of parasite maturation during blood-stage *Plasmodium* infection. Proc Natl Acad Sci USA.

[10] Apte SH, Groves PL, Roddick JS, da Hora VP, Doolan DL (2011). High-throughput multi-parameter flow-cytometric analysis from micro-quantities of *Plasmodium*-infected blood. Int J Parasitol.

[11] Jimenez-Diaz MB, Ebert D, Salinas Y, Pradhan A, Lehane AM, Myrand-Lapierre ME (2014). (+)-SJ733, a clinical candidate for malaria that acts through ATP4 to induce rapid host-mediated clearance of *Plasmodium*. Proc Natl Acad Sci USA.

[12] Croft SL, Duparc S, Arbe-Barnes SJ, Craft JC, Shin CS, Fleckenstein L (2012). Review of pyronaridine anti-malarial properties and product characteristics. Malar J.

[13] Gaur AH, McCarthy JS, Panetta JC, Dallas RH, Woodford J, Tang L (2020). Safety, tolerability, pharmacokinetics, and antimalarial efficacy of a novel *Plasmodium falciparum* ATP4 inhibitor SJ733: a first-in-human and induced blood-stage malaria phase 1a/b trial. Lancet Infect Dis.

[14] Deng X, Duffy SP, Myrand-Lapierre ME, Matthews K, Santoso AT, Du YL (2015). Reduced deformability of parasitized red blood cells as a biomarker for anti-malarial drug efficacy. Malar J.

[15] Zhang R, Suwanarusk R, Malleret B, Cooke BM, Nosten F, Lau YL (2016). A basis for rapid clearance of circulating ring-stage malaria parasites by the spiroindolone KAE609. J Infect Dis.

[16] Okoth WA, Dukes EJ, Sullivan DJ (2018). Superior pyronaridine single-dose pharmacodynamics compared to artesunate, chloroquine, and amodiaquine in a murine malaria luciferase model. Antimicrob Agents Chemother.

[17] Pascual A, Parola P, Benoit-Vical F, Simon F, Malvy D, Picot S (2012). Ex vivo activity of the ACT new components pyronaridine and piperaquine in comparison with conventional ACT drugs against isolates of *Plasmodium falciparum*. Malar J.

[18] Tona Lutete G, Mombo-Ngoma G, Assi SB, Bigoga JD, Koukouikila-Koussounda F, Ntamabyaliro NY (2021). Pyronaridine-artesunate real-world safety, tolerability, and effectiveness in malaria patients in 5 African countries: a single-arm, open-label, cohort event monitoring study. PLoS Med.

[19] West African Network for Clinical Trials of Antimalarial D (2018). Pyronaridine-artesunate or dihydroartemisinin-piperaquine versus current first-line therapies for repeated treatment of uncomplicated malaria: a randomised, multicentre, open-label, longitudinal, controlled, phase 3b/4 trial. Lancet.

[20] Lundie RJ, de Koning-Ward TF, Davey GM, Nie CQ, Hansen DS, Lau LS (2008). Blood-stage *Plasmodium* infection induces CD8^+^ T lymphocytes to parasite-expressed antigens, largely regulated by CD8alpha^+^ dendritic cells. Proc Natl Acad Sci USA.

[21] Haque A, Best SE, Unosson K, Amante FH, de Labastida F, Anstey NM (2011). Granzyme B expression by CD8^+^ T cells is required for the development of experimental cerebral malaria. J Immunol.

[22] Cromer D, Evans KJ, Schofield L, Davenport MP (2006). Preferential invasion of reticulocytes during late-stage *Plasmodium berghei* infection accounts for reduced circulating reticulocyte levels. Int J Parasitol.

[23] Haque A, Best SE, Amante FH, Ammerdorffer A, de Labastida F, Pereira T (2011). High parasite burdens cause liver damage in mice following *Plasmodium berghei* ANKA infection independently of CD8(+) T cell-mediated immune pathology. Infect Immun.

[24] Haque A, Best SE, Amante FH, Mustafah S, Desbarrieres L, de Labastida F (2010). CD4^+^ natural regulatory T cells prevent experimental cerebral malaria via CTLA-4 when expanded in vivo. PLoS Pathog.

[25] Auparakkitanon S, Chapoomram S, Kuaha K, Chirachariyavej T, Wilairat P (2006). Targeting of hematin by the antimalarial pyronaridine. Antimicrob Agents Chemother.

